# Cytoscape.js: a graph theory library for visualisation and analysis

**DOI:** 10.1093/bioinformatics/btv557

**Published:** 2015-09-28

**Authors:** Max Franz, Christian T. Lopes, Gerardo Huck, Yue Dong, Onur Sumer, Gary D. Bader

**Affiliations:** The Donnelly Centre, University of Toronto, Toronto, ON M5S 3E1, Canada

## Abstract

**Summary:** Cytoscape.js is an open-source JavaScript-based graph library. Its most common use case is as a visualization software component, so it can be used to render interactive graphs in a web browser. It also can be used in a headless manner, useful for graph operations on a server, such as Node.js.

**Availability and implementation:** Cytoscape.js is implemented in JavaScript. Documentation, downloads and source code are available at http://js.cytoscape.org.

**Contact:** gary.bader@utoronto.ca

## 1 Introduction

Network information in biology continues to grow in utility in many contexts, from analysis of cellular mechanisms to identifying disease biomarkers. Further, the web is increasingly a platform for apps with complex user interfaces that use standard technologies such as HTML, CSS and JavaScript (JS). Cytoscape.js provides a JS application programming interface (API) to enable software developers to integrate graphs into their data models and web user interfaces. Cytoscape.js can be used in several domains, such as biological networks or social graphs. Cytoscape.js is the modern replacement for the Adobe Flash-based Cytoscape Web (Lopes *et al.*, 2010).

## 2 Implementation

Cytoscape.js is implemented as a standalone JS library. It has no hard dependencies; neither browser plugins nor other libraries are required. However, it includes hooks to several useful libraries and environments, including CommonJS/Node.js, AMD/Require.js, jQuery, Bower, npm, spm and Meteor. This allows Cytoscape.js to integrate into a wide variety of JS-based software systems.

**Fig. 1.**
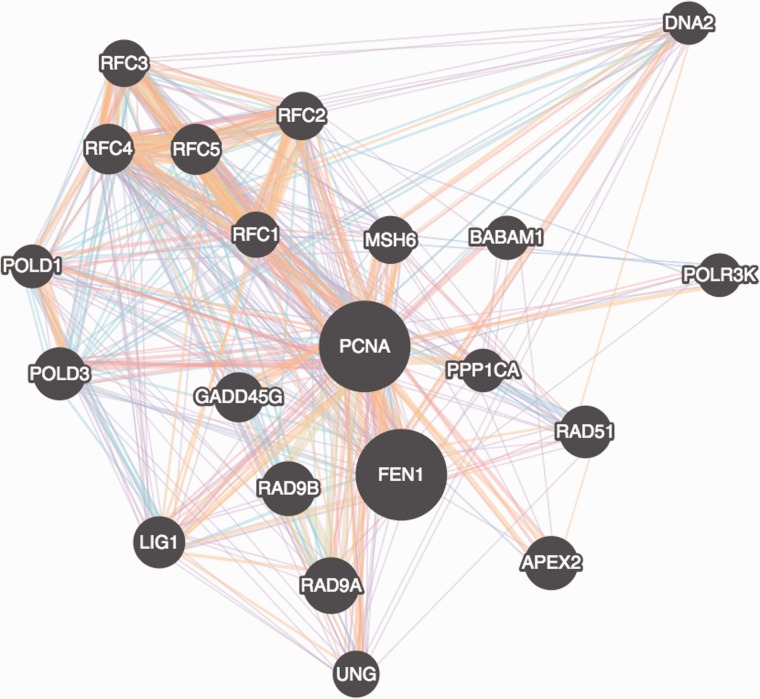
A GeneMANIA gene–gene interaction network automatically laid out and visualised with Cytoscape.js, showing interaction strength (edge thickness), interaction type (colour), multiple edges between nodes, protein score (node size) defined using a stylesheet

The architecture of Cytoscape.js allows it to be run headlessly (i.e. without a graphical user interface) or as a visualisation component (Fig. 1), using HTML5 canvas as its underlying implementation. This allows Cytoscape.js to be run on both the client side—i.e. the browser—and the server-side, an important consideration as JS code is increasingly being shared with the client and the server.

For increased ease of use, the library shares several concepts with the HTML + CSS + JS web model. Styling in Cytoscape.js is specified using CSS-like stylesheets, sharing as much syntax as possible with CSS. Similarly, graph elements are analogous to HTML DOM elements—they are styled by the stylesheets and programmatically accessible via the JS core API.

The Cytoscape.js architecture is composed of the core and the collection. The core is a developer’s main entry point into the library. It represents the graph and is used to run layouts, alter the view, and perform other operations on the graph as a whole. Core functions are available to access graph elements. Each of these functions returns a collection, a set of graph elements. A collection has its own API that can be used to filter, traverse, perform operations on and get data about the elements in the collection. Some core functions take collections as input.

## 3 Features

### 3.1 Feature set

Cytoscape.js features include, but are not limited to, the following:

*Graph types*: Cytoscape.js supports several types of graphs, including traditional graphs, directed graphs, undirected graphs, multigraphs and hypergraphs (with compound nodes, but not yet with hyperedges).

*Mutable graphs*: The graph can be manipulated by adding, removing, or modifying the state of graph elements. This enables apps to provide highly interactive graphs for the user.

*Graph traversal*: Graph traversal functions are provided, which are useful for both user interface interactions and programmatic graph analysis.

*Graph theory algorithms*: Several well-known graph theory algorithms—such as connectivity search, shortest path, minimum spanning tree, minimum cut, ranking and centrality measures—are included.

*Stylesheets*: Stylesheets are used to specify the visual style of elements. Selectors and classes are supported in order to map element state to style. Similar to the Cytoscape desktop app (Shannon P. *et al.*, 2003), a functional mapper syntax is provided to map particular style properties based on element data—e.g. node colour mapped from a numerical weight. Stylesheets can be replaced at runtime, changing the overall visual style of the graph.

*Built-in gesture support for mouse and touch based devices*: The default Cytoscape.js renderer supports the touch and mouse gestures a user would expect out-of-the-box. Nodes can be dragged. The user can manipulate the viewport with gestures like pinch-to-zoom and drag-to-pan.

*Event binding*: Events can be bound in several ways. Delegation can be used such that newly added elements trigger bound events. Bindings can be added and removed, and the multiplicity of elements-to-triggered-events (i.e. for which elements and how many times the handler should be triggered) can be specified when binding. Several higher-level events are provided such that the type of user interaction is abstracted, e.g. ‘tap’ works both with computer mice and finger presses on touch devices.

*Animations*: Animations can be used to increase the salience of particular elements in the graph and to provide visual continuity to the user when programmatic changes to the graph are made.

*Compound nodes*: As an addition to the traditional graph model, compound nodes are a way for the developer to embed nodes within another node. Compound nodes are useful for representing things like biological complexes and their subunits.

*Import & export*: The graph can be exported as an image (PNG or JPG), including at high resolution for publication. Cytoscape.js supports importing and exporting graphs via JSON, thereby allowing for full serialisation and deserialization of graph state.

*Layouts*: Layouts provide a mechanism for automatically positioning nodes in a graph. Alternatively, the developer may specify pre-determined node positions. Default layouts include null, random, preset, grid, circle, concentric, breadthfirst, dagre, cose, cola, spread, arbor and springy.

*Extensibility*: Cytoscape.js provides mechanisms for the developer to extend its behaviour. For instance, user interface widgets can be built on top of the library—several of these extensions, including contributed layout algorithms, exist. Layouts, core and, collection functions and renderers can be written as extensions to add to the library without needing to modify it directly.

### 3.2 Performance

Cytoscape.js can comfortably render thousands of graph elements on average hardware. Rendering performance is affected by the visual styles used, the graph size and the web browser client. Rendering performance can be improved by using simpler styles—especially for edges. Optional features that improve the real and user-perceived large graph interaction performance are detailed in the online documentation.

Cytoscape.js is frugal with respect to rendering: A new frame is rendered only when the view needs to be updated. So, a developer can safely use the API for analysis without worrying about overhead caused by rendering. There is no rendering overhead when using Cytoscape.js headlessly.

### 3.3 Documentation

Cytoscape.js is actively developed as an open-source project, and is freely available at http://js.cytoscape.org. The documentation includes an in-depth description of the API, runnable code examples and demos.

### 3.4 Example applications

Examples of Cytoscape.js use include the ‘Export network as a web page’ feature of the Cytoscape desktop application, ConsensusPathDB (Kamburov A *et al.*, 2013), InnateDB (Breuer K *et al.*, 2013), BioJS library components (Corpas M, 2014), the *Saccharomyces* genome database (Costanzo MC *et al.*, 2013), the RCyjs Bioconductor package (http://www.bioconductor.org/packages/ release/bioc/html/RCyjs.html), the upcoming release of GeneMANIA (Warde-Farley *et al.*, 2010), cyNetShare (https://idekerlab.github.io/cy-net-share/), NDEx (http://www.ndex bio.org/), Elsevier (http://www.elsevier.com/books-and-journals/content-innovation/cytoscape) and demos linked to on the Cytoscape.js site (http://js.cytoscape.org/).

### 3.5 Future directions

We intend to add new visual styles, extensions, graph analysis APIs and layouts, as well as improve the extension ecosystem and increase performance. We encourage user community feedback to elicit new library features.
